# Tele–Robotic Platform for Dexterous Optical Single-Cell Manipulation

**DOI:** 10.3390/mi10100677

**Published:** 2019-10-08

**Authors:** Edison Gerena, Florent Legendre, Akshay Molawade, Youen Vitry, Stéphane Régnier, Sinan Haliyo

**Affiliations:** 1 Institut des Systèmes Intelligents et de Robotique, ISIR, Sorbonne Université, CNRS, F-75005 Paris, France; 2 TIPS Laboratory, CP 165/67, Université libre de Bruxelles, 50 Avenue F. Roosevelt, B-1050 Brussels, Belgium

**Keywords:** cell manipulation, 3D cell rotation, mobile micro-robots, optical tweezers, tele-robotics

## Abstract

Single-cell manipulation is considered a key technology in biomedical research. However, the lack of intuitive and effective systems makes this technology less accessible. We propose a new tele–robotic solution for dexterous cell manipulation through optical tweezers. A slave-device consists of a combination of robot-assisted stages and a high-speed multi-trap technique. It allows for the manipulation of more than 15 optical traps in a large workspace with nanometric resolution. A master-device (6+1 degree of freedom (DoF)) is employed to control the 3D position of optical traps in different arrangements for specific purposes. Precision and efficiency studies are carried out with trajectory control tasks. Three state-of-the-art experiments were performed to verify the efficiency of the proposed platform. First, the reliable 3D rotation of a cell is demonstrated. Secondly, a six-DoF teleoperated optical-robot is used to transport a cluster of cells. Finally, a single-cell is dexterously manipulated through an optical-robot with a fork end-effector. Results illustrate the capability to perform complex tasks in efficient and intuitive ways, opening possibilities for new biomedical applications.

## 1. Introduction

Dexterous manipulation of single-cells offers many possible applications in cellular surgery, mechanobiology, tissue engineering, and biophysics. Recent breakthroughs in biotechnology are rising the demand for complex single-cell operation techniques such as cell isolation, 3D orientation, and cell-injection. Nowadays, those tasks are usually performed using simple three-axis cartesian robots consisting of motor-driven micromanipulators with prismatic joints and equipped with micro-pipettes or micro-grippers as end-effectors. The operator directly controls a single actuator through buttons or knobs, ignoring the overall kinematics of the robot. Basic tasks, like the rotation of a cell, are proven to be quite time-consuming and challenging due to the lack of dexterity of those micromanipulators and their control interfaces. Consequently, those devices have a steep learning curve.

Significant challenges remain for applications related to single-cell manipulation mainly due to the physics involved (volumetric forces dominated by the surface forces) and the size limitations imposed by the environment. The resolution and precision required at those sizes have a cost in terms of degrees-of-freedom, workspace, grasping strategies and control schemes. Furthermore, there is an increasing demand to manipulate objects in confined environments like micro-fluidic devices, in order to decrease flow disturbances, contamination or evaporation of the culture medium, rendering external actuators unusable. All these constraints call to replace current techniques by non-contact manipulation methods.

Accordingly, a great effort has been made in the search of solutions for the actuation of mobile microrobots (i.e., untethered robots where the entire body is micrometer-sized) to serve as remote manipulators. A variety of methods have been developed employing chemical reactions, physical fields or bio-hybrid approaches [1,2]. Remote actuation using different external energy-fields like magnetic, acoustic or optical has appeared as a very promising solution in applications where high spatial maneuverability and precision are required [3]. Among them, optical trapping [4] (awarded with the Nobel Prize in physics 2018) offers several advantages in the manipulation of small biological samples [5].

Optical manipulation exploits the light radiation pressure to noninvasively trap and position suspended micro-objects and cells with a nanometer resolution; resulting in a contamination-free, contact-free, and label-free method for cell manipulation in their original culture medium. Their compatibility with other optical techniques, especially microscopy implies that these are highly appropriate for lab-on-chip systems and micro-fluidic devices. In addition, it is possible to simultaneously trap several objects using a unique laser source by active diffractive optical elements (holographic optical tweezers) [6] or by rapid laser-deflection (time-shared methods) [7]. Furthermore, optical-robots (i.e., 3D-printed micro-structures actuated using laser trapping techniques) can be used to indirectly handle the cells in applications where their viability is an important issue. Physical and chemical treatments in optical-robots also allow functionalization for more specific tasks such as pH sensor [8], temperature sensor [9], puncturing of cells [10] or syringe function [11].

Although recent few examples of automated direct cell-rotation [12] or automated cell transportation through microtool [13] have been proposed, automation at micro-scales remains a very challenging issue in most cases where working conditions are uncertain and samples unstructured. Automating a specific task is a time-consuming operation, often beyond the skills of the end-user. As optical-robots can be directly 3D printed according to the needs of a given experiment, each structure will have its own characteristics, and the system must be able to adapt to this new tool. Also, most tasks require some human know-how as the operator can determine the optimal method or protocol depending on the use case.

Similar issues in macro-manipulation robotics have been treated using teleoperated schemes, where a master device is handled by the operator to control a slave robot. This approach integrates human intelligence in the robotic control loop, allowing user’s expertise and ability to adapt the manipulation protocol to environmental disturbances and the peculiarities of the task. A very well-known example is the Da Vinci System (Intuitive Surgical, Inc.) for robotic surgery. It is designed to facilitate complex minimally invasive surgery and it is controlled by a surgeon from a console. Tele–robotics have also been successfully implemented in a large range of applications where the operator is unable to interact directly with the environment, like underwater vehicles or nuclear robots.

Existing commercial interfaces for optical manipulation allow the user to control the optical traps using 2D mouse position. In order to enhance the user control, some attempts to incorporate more efficient master devices have been made, such as joysticks [14], gesture recognition (e.g., 2D cameras, kinect, leap-motion) [15,16,17], multi-touch tablets [18,19] and 3D robotized interfaces (or haptic interfaces) [20,21,22]. Multi-modal approaches have also been presented with a combination of gesture recognition, eye gaze tracking or speech recognition [23,24]. Despite the improvement brought by those attempts in terms of ergonomy and efficiency, completing complex real-world tasks still remains a challenge. Mouses, joysticks, and tablets do not permit a three-dimensional workspace. Tracking sensors suffer from low temporal and spatial resolution. As 3D robotized interfaces have only been used to manipulate one particle at a time, limiting the scenarios in which the number of objects exceeds the abilities of one operator. In addition, multi-trap actuation techniques used on most of these platforms are based on spatial light modulators (SLM), which suffers from high time latency resulting from the reactivity of their hardware and a high computational cost for a given trajectory.

Robust 3D real-time micro-manipulation requires high spatial and temporal resolution, as the end-effector interacts in a micro-world with high dynamics effects. Working-space, degrees-of-freedom (DoF), immersion, and flexibility are also essential characteristics of an efficient micro-manipulator. Based on all these observations, we propose a teleoperated optical-micromanipulation platform for direct and indirect single-cell dexterous manipulation. The content of this paper has been partially mentioned in a previous conference paper [25]. This article presents a more detailed methodology and additional studies on the precision and efficiency of the proposed platform. Further, two new results are reported: the direct and indirect manipulation of a single erythrocyte, a suspended red blood cell.

The system is based on optical actuation, allowing a non-contact manipulation of biological samples or micro-machines. The workspace is optimized by combining a 3D multi-trap time-shared method, a 3D nano-stage, and a 2D micro-stage. To solve the latency issues, the implemented 3D multi-trap technique is based only on high-bandwidth steering mirrors [7]. Teleoperation control is implemented with a master device, Omega.7, providing 6+1 DoF and is ensured by a hard real-time system. Traps can be grouped and controlled in a variety of ways for specific purposes enlarging the DoF of the slave device. The performance of the system in terms of static and dynamic precision is evaluated using trajectory control tasks. Three state-of-the-art experiments have been performed to verify its efficiency. First, the reliable 3D rotation of a cell has been demonstrated. Secondly, the transport of a cluster of cells has been performed with an optical-robot. Finally, a single-cell has been dexterously manipulated using an optical-robot with a fork end-effector. These results illustrate the kind of complex biomedical applications that can be effectively and intuitively accomplished with the proposed platform.

## 2. Materials and Methods

### 2.1. Teleoperation System Design

**Figure 1:**
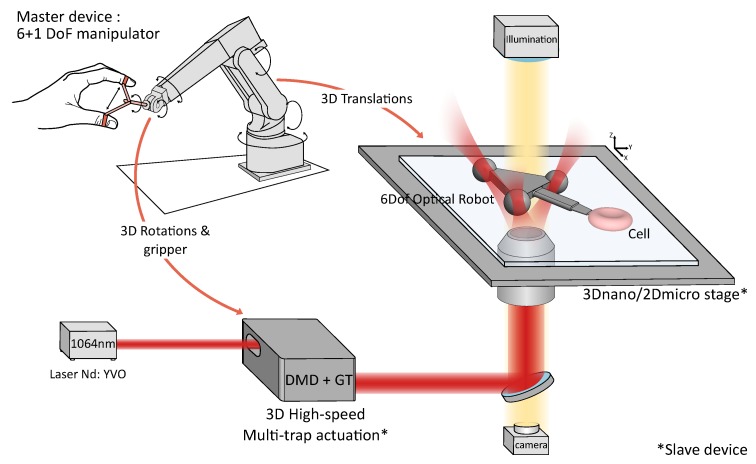
Optical-micromanipulation platform for dexterous single-cell handling.

The platform was composed of three main parts: the optical set-up (Laser and passive optical components forming the light path), the slave robot (robot-assisted stages and active optical components for 3D multi-trap actuation), and the master device (seven-DoF robot manipulator). Figure 1 shows a schematic representation of the platform. The system can be used to manipulate biological samples directly or indirectly (i.e., through trapped inert objects).

#### 2.1.1. Optical System

The system is constructed around a custom made inverted microscope. The microscope objective (oil immersion, Olympus UPlanFLN 40x, NA 1.3) was used to visualize the sample and to generate the optical-traps. A near-infrared laser (1070 nm) has been chosen as source to minimize biological damages. The beam is expanded in order to overfill (20%) the objective entrance, thus improving the trapping efficiency [26]. The illumination (LED, 3 W) is reflected by a longpass dichroic mirror into a high-speed CMOS camera (Basler, Ahrensburg, Germany, 659 × 494 px) to provide visual feedback and environmental information to the operator.

#### 2.1.2. Slave Robot: Robot-Assisted Stages and High-Speed 3D Multi-Trap Actuation

Two different types of actuation coexist to control the motion of optical traps. The first one is composed of a 3D nano-stage mounted on a 2D micro-stage. These nano-stage and micro-stage move the sample chamber while trapped objects remain fixed. The micro-stage allows for a large workspace (25 × 25 $mm^{2}$), while the nano-stage gives a finer control in 200 $\mu m^{3}$ range.

The second actuation uses high-speed laser-deflection generated by a galvanometer (GVS002, Thorlabs, Newton, NJ, USA) and a deformable mirror (PTT111 DM, Iris AO, Berkeley, CA, USA). The 3D motion of the focal spot is obtained by the synchronization of the orientation of the galvanometer mirror and focusing or defocusing the deformable mirror. Multiple traps are hence created by sequentially moving the focal spot between different positions. This time-sharing method is made possible by the short response time of the galvanometer and the deformable mirror, and a hard real-time control framework implemented in C++ on a Real-Time kernel (Xenomai). This design allows the creation of numerous independent optical traps within a volume of approximately 70 × 50 × 9 $\mu m^{3}$ with a bandwidth up to 200 Hz. This technique is aberration-free as it uses only mirrors with high reflectance and is designed to generate equally efficient and stable traps regardless of their position in the workspace. For further details please see [7].

#### 2.1.3. Master–Slave Coupling

The master device is an Omega (Omega.7, ForceDimension), which allows seven-DoF. On the seven-DoF, three-DoF are for translations, three-DoF are for rotations and a last DoF is given by a gripper under the index finger of the user. The work-space is 160 × 160 × 110 $mm^{3}$ for translation, 240 × 140 × 180 deg for rotations and 25 mm for grasping.

The master device translation is appropriately transformed (scaling factor of $\times 10^{-4}$) and sent to the nano-stage. The orientation of the master device is used to compute a rotation matrix which indicates trap positions to the galvanometer and to the deformable mirror. The rotation matrix is calculated using the Tait–Bryan angles with z−y−x convention (yaw, pitch and roll). Finally, the gripper position is interpreted to determine new trap positions depending on the configuration. The gripper has two operating modes. The radial mode moves the desired traps toward or away from the rotation center. This type of radial motion allows the user to grasp objects using trapped beads as ‘fingers’. The scissor mode rotates the wanted traps towards the Y-axis in order to give a scissor-like movement to a set of traps. It can be used to actuate a tool such a clamp.

**Figure 2:**
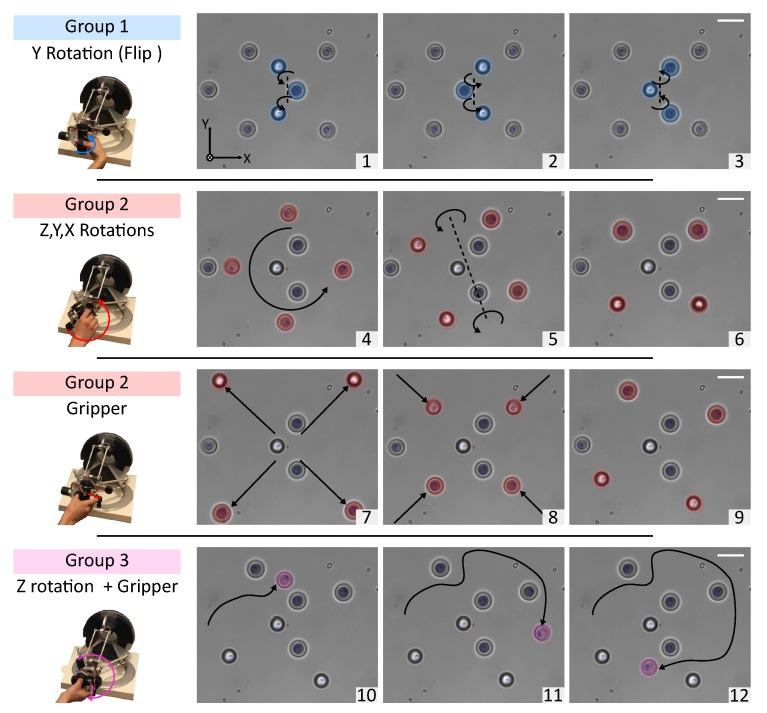
Example of 3D teleoperation using three groups of traps. The operation is a succession of four different tasks demonstrating the system’s capabilities. The first task (1, 2 and 3) is a flip around the y-axis of three micro-beads. The second task (4, 5 and 6) shows the 3D control of four micro-beads. The third task (7, 8 and 9) presents the ‘radial’ operating mode of the gripper. Finally, the last task (Picture 10, 11 and 12) presents the travel of one micro-bead around all the others. Scale bars are 5 μm long.

The optical traps can be dynamically created via the control interface. Every trap position can be directly edited in the micro-world coordinates. The traps can also be organized by groups. For each group, the 3D rotations, the 3D translations and the gripper functionality can be independently enabled or disabled. The number of traps is virtually unlimited; however as the stiffness decreases with the number of traps, the amount of stable traps in dynamic and static configuration is around 15 and 30 respectively. Figure 2 shows an example of 3D tele-operation using three groups of traps.

Translations and rotations have two control modes:

1. Position Control: This first mode mirrors the master device’s position and orientation to the slave robot, with an appropriate scaling factor. This factor can be chosen according to the task’s dimensions and the operator’s comfort. This method is suitable to execute precise tasks.
2. Velocity Control: This second mode enables control of the slave robot’s velocity. The motion’s direction and amplitude are computed according to the vector made by the center of the master device’s workspace and the position of the handle. A scaling factor can also be chosen according to the task’s requirements. A maximal handling velocity is also defined according to the number of traps, in order to assist the user and help the trapped objects’ retention. The velocity control mode can be enabled independently for translations and rotations, and is suitable for long displacements like sample chamber exploration or for continuous rotation of an object like micro-pump [27] or cell rotation for tomographic imaging [28].

### 2.2. Evaluation of Teleoperation Performances

To evaluate the system’s performances, several experiments with micro-beads have been carried out. Polystyrene beads have been chosen, as they are the most common tool used in optical tweezers manipulation systems.

Data from the master device, the set-points of actuators, and video images at 64 fps from the CMOS camera are recorded during different tasks. Each data record contains the current system time (Xenomai timer) for synchronization purposes. Measured positions of the trapped beads are extracted in an off-line process using the circle hough transform algorithm from OpenCV software. The image has a resolution of 659 × 494 px and cover a surface of 70 × 50 μm and the theoretical resolution of the tracking algorithm is 2 px, corresponding to about 200 nm. As the 2D image tracking algorithm does not permit to estimate the depth, only 2D projection of the 3D motions are studied. Same laser power (400 mW), trap irradiation time (5 ms), and polystyrene micro-beads (3 μm diameter, refractive index ∼1.59) were used for all tasks.

For translations motions, nano-stage (P-562.3CD, Physik Instrumente, Karlsruhe, Germany) and piezo-controller (E-725.3CD, Physik Instrumente, Karlsruhe, Germany) are used in closed-loop with a resolution of 1 nm, 20 kHz sampling rate and factory calibration. For the sake of simplicity, only rotational motions are considered here.

#### 2.2.1. Static Precision

The static precision depends on the actuators’ accuracy, thermal noise, and trap stiffness. The deformable mirror is controlled in open-loop based on its calibration and has nanometer and microradian resolution (manufacturer data: wavefront resolution <15 nm rms). The galvanometer is controlled in closed-loop with an angular resolution of 15 μrad.

In time-sharing scenarios, when more than one trap is created, the laser switches from trap to trap. In this case, each bead is only held by the laser during a fraction of the cycle, and the rest of the time the micro-bead is subjected to Brownian fluctuations and other environmental forces [29]. In consequence, the effective stiffness of each trap is diminished by the reduced duty cycle and the precision of the bead’s position is inversely related to the number of traps created.

**Figure 3:**
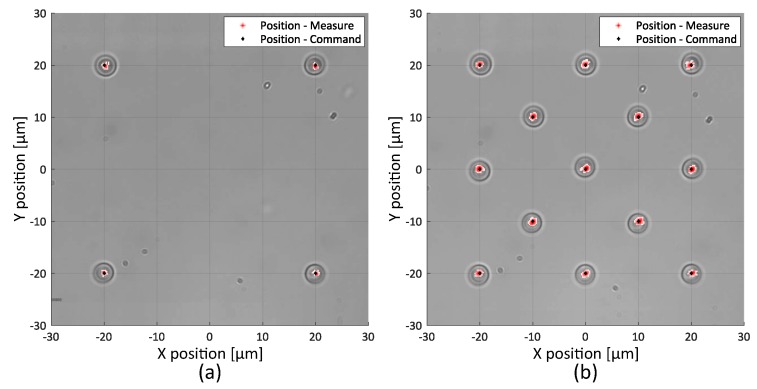
Static precision tasks (**a**) Four micro-beads positioned in a square of 40 × 40 μm. The maximum error between the command and the actual position is 400 nm. (**b**) Thirteen micro-beads set to cover a 40 × 40 μm square. The maximum position error is up to 1 μm and is essentially due to Brownian motion. Positions are measured during 15 s.

Different static tasks have been performed and recorded during 15 s. The static position error value is computed as the maximum error between the trap’s position command and the bead’s tracked position. For a single trap, no differences between the trap’s command position and bead’s measured position are detected, as the stiffness of the trap highly reduced the Brownian fluctuations. We conclude that the error position is less than the resolution of the tracking algorithm, i.e., the position’s maximum error is less than 200 nm. For four and thirteen micro-beads positioned in a square of 40 × 40 μm, the position error is calculated as 400 nm and 1 μm respectively, and is essentially due to Brownian motion as the effective stiffness of each trap decreases as the number of traps increases. These experimental results for four and thirteen traps are presented in Figure 3.

#### 2.2.2. Trajectory Control

**Figure 4:**
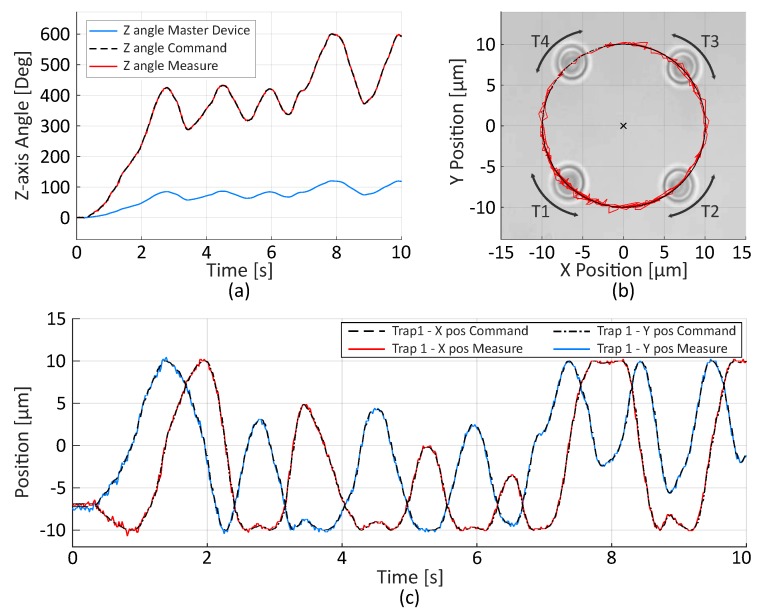
Example of teleoperated rotation in position mode of a group of four micro-beads. (**a**) The master device orientation and the command sent to the actuators. Z angle is the angle of rotation around the z-axis. The scaling factor is set to 5 in order to allow a complete rotation. The measured angle is estimated from the measured position of Trap T1. (**b**) The 2D trajectory followed by the Trap T1. (**c**) The measured position of Trap T1 computed from the video and the set-point. Estimated error is 0.31 μm with a standard deviation of 0.23 μm.

Three different tasks have been performed to evaluate the trajectory control. First, four micro-beads have been trapped and rotated in position mode. The master device orientation is used to compute the optical trap position. The scaling coefficient is set to 5 in order to achieve complete rotations of the trapped particle. The measured position of the Trap T1 and the master trajectory are shown in Figure 4. The mean error between command and real position is 0.31 μm with standard deviation of 0.23 μm mainly due to Brownian Motion.

**Figure 5:**
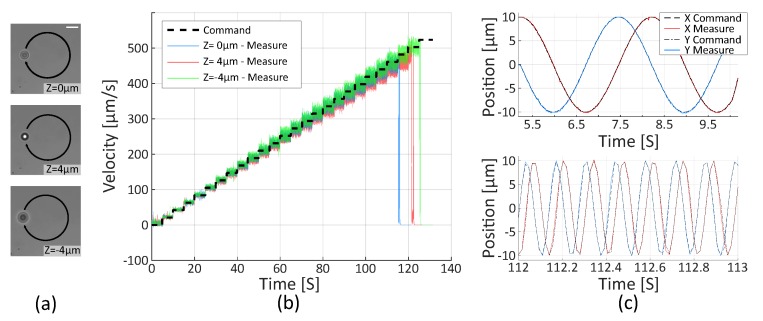
Teleoperated rotation in velocity mode of one micro-bead at different Z positions. (**a**) Pictures of a trapped bead in three different axial configurations. Scale bar is 5 μm long. (**b**) Velocity reference and measured velocity in three different configurations (**c**) X and Y positions of the Trap 1 in “Z = 0 μm” configuration at a speed of 21 μm/s and 462 μm/s. The maximal reachable velocity without losing the traps is 462 μm/s with an error of 4%.

The second task consists in trapping a micro-bead at different axial positions and rotates it in velocity mode, increasing the speed by steps of 21 μm/s. The handle orientation of the master device is locked to generate speed steps using an adjustable command gain. Positions of micro-beads are extracted off-line from video images using a circle tracking algorithm and the velocity is computed as the discrete derivative of the position. Results shown in Figure 5 confirm that the micro-bead accurately follows the velocity reference from 21 μm/s to 462 μm/s. Finally, the bead is lost at 483 μm/s.

**Figure 6:**
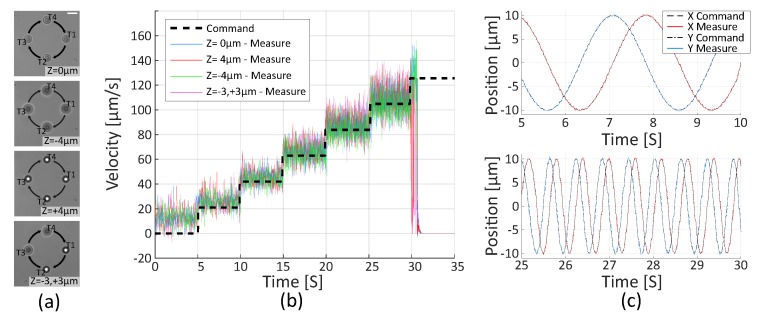
Teleoperated rotation in velocity mode of four micro-beads at different Z positions. (**a**) Pictures of four trapped beads in different axial configurations. Scale bar is 5 μm long. (**b**) Velocity reference and measured velocity in 4 different configurations. (**c**) X and Y positions of the Trap 1 in “Z = 0 μm” configuration at a speed of 21 μm/s and 105 μm/s. The maximal reachable velocity without losing the traps is 105 μm/s with an error of 5%.

The same experiment performed with a group of four trapped micro-beads with different axial configurations, shown in Figure 6, also demonstrates proper following of the references. The maximal reachable velocity without losing the beads are 105 μm/s. Since the beads are lost at the same speed in the different configurations, these experiments validate the proposed velocity control and corroborate that the system produces equally efficient and stable traps, regardless of the traps’ 3D positions.

#### 2.2.3. Velocity Limitation

As expected, the escape velocity to release the trapped bead is higher for one trap than for four traps. The laser is deflected at a constant frequency of 200 Hz from one trap to another, meaning that the position is moving by steps, and greater velocities imply larger steps. The velocity thresholds estimated in Section 2.2.2 are 462 μm/s for one trap and 105 μm/s for four traps.

For both experiments, the velocity threshold corresponds to a position step of approximately 2.3 μm. Same results are found with two, three, five and nine traps. Hence, this step-size defines the limit before risking to free a trapped object.

The following equation predicts the theoretical highest reachable velocity depending on the number of optical traps generated and the deflection frequency of the laser:

(1) $$V_{max}=\frac{D_{max}*f}{N_{trap}},$$

where $V_{max}$ is the velocity threshold, $D_{max}$ the position step-size, $N_{trap}$ the number of optical traps, and *f* the deflecting frequency of the laser. As a consequence, if the deflecting frequency of the laser is set to a higher value, the velocity threshold increases.

In regard to the translation actuation, since the whole sample-chamber is moved, if only one trap is generated the velocity threshold is only limited by the drag force, as the trap is always active. The escape velocity is measured at 1500 μm/s, resulting in an escape force of 38 pN. When several traps are generated, the velocity threshold is limited by the deflecting frequency and the viscous drag forces. During the time period when the trap is not active, viscous forces will shift the object out of the trap. If the motion is small enough, the bead will be attracted to equilibrium position when the trap is activated back. However, when the displacement of the bead is large enough during a period (i.e., if the translation velocity is too high) the bead is not in the attractive zone anymore when the trap is active again. For two, three, four and five traps, the velocity threshold measured is respectively 300 μm/s, 220 μm/s, 110 μm/s and 80 μm/s. Note that escape forces are defined by optical properties at the very edges of the trap, where the restoring force is no longer a linear function of the displacement [30]. Further investigations on the effective stiffness of traps combining time-sharing actuation and stage-based actuation is required for a higher precision on force measurements. Table 1 summarizes the principal parameters of the system.

To illustrate the kind of biological applications that can be accomplished with the proposed platform in a real world scenario, direct and indirect manipulations of mouse erythrocytes, suspended red-blood cells (RBCs), are presented in the next section.

## 3. Results and Discussion

### 3.1. Direct Manipulation: 3D Rotation of a Cell

Presently, 3D orientation of biological samples has gained much attention due to its involvement in various single-cell surgeries and cell imaging techniques. Accordingly, significant efforts have been made toward achieving 3D cell orientation control using holographic optical tweezers (HOT) [12,31,32,33]. Although these SLM-based techniques represent elegant solutions for the 3D rotations, the intrinsically slow response of liquid crystal and the complexity of trajectory computation induce important delays making their implementation in real-time scenarios a difficult task even when only reduced to two traps. In time-sharing techniques, one of the main advantages is that the control of traps is straightforward, requiring only the 3D transformation that produces the desired movement and its direct conversion into actuator coordinates.

The first experiment is dedicated to the 3D orientation of a suspended erythrocyte. A group of eight traps arranged along the perimeter of the cell has been created through the user interface. Initially, the erythrocyte was sedimented on a coverslip with face-on orientation to the optical axis. The eight traps enable the rotation of the cell with respect to the x, y, and z axes, and the simultaneous translation in all three axes. Thanks to the high bandwidth and the efficiency of the proposed system, the user can control the 3D motion of the cell without any noticeable latency.

**Figure 7:**
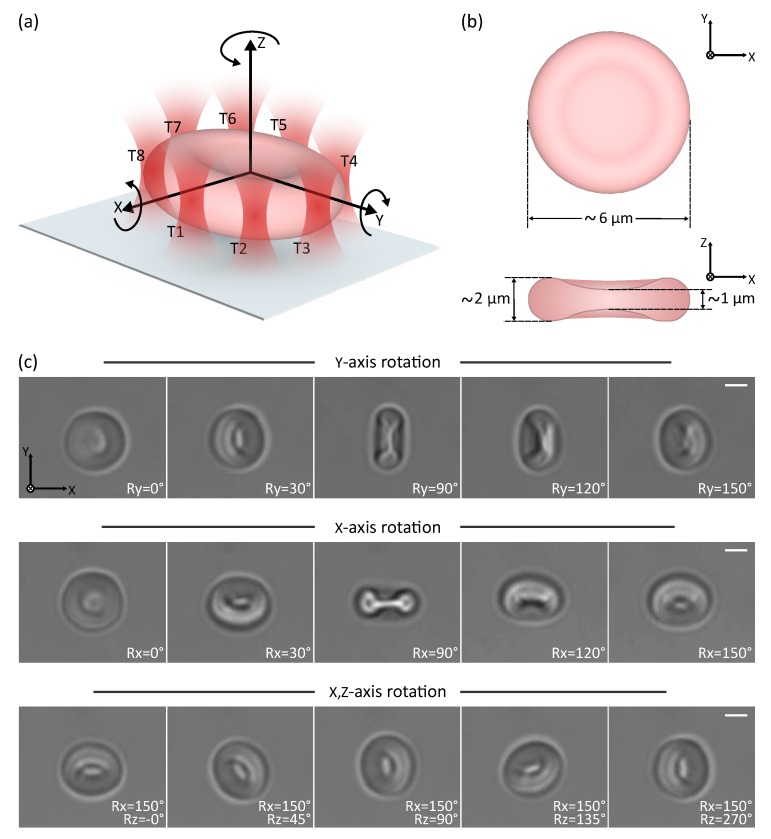
3D rotation control of erythrocyte driven by 8 optical traps. (**a**) Schematic 3D depiction of erythrocyte and the reference frame used in the 3D control. (**b**) Schematic 2D depiction of the erythrocyte with dimensions in y–x and z–x plane. (**c**) Time-lapse images from Supplementary Videos S1 and S2 demonstrating multiple degrees-of-freedom cell rotational control. Scale bar is 2 μm long.

Figure 7 and Supplementary Video S1 show the six-DoF control of an individual cell. Every DoF can be controlled independently or coupled to another. Rotation’s scaling factor is set to three in order to achieve full 360${}^{\circ }$ rotation. The maximum velocity before observing minor overshooting is 188.6 deg/s or 9.92 μm/s for a cell with 6 μm diameter. The difference between the theoretical maximum velocity for eight micro-beads (57.5 μm/s) and the measured velocity for RBCs may be due to the lower cell’s refractive index (RI ∼ 1.38), the higher viscous drag torque due to larger surfaces, and the shape differences. Factors such as symmetry, size, and non-homogeneity of manipulated objects will impact the performance of direct manipulation.

Rotation can also be controlled in speed mode, in order to allow constant displacement of a cell (see Supplementary Video S2). True 360${}^{\circ }$ rotation around all-axes of a single RBC is successfully demonstrated. The achievable orientation range depends on the maximum distance separating the traps and the center of rotation and will be limited by the workspace for bigger cells.

### 3.2. Indirect Manipulation: Six-DoF Teleoperated Optical Robot for Cell Manipulation

Direct optical manipulation is the simplest and most used manipulation technique; however, several studies have shown that direct laser exposure can cause considerable photo-damage [34,35]. Furthermore, it is difficult to reliably hold different kinds of biological samples as the stability of the traps depends on the shape, material, and the refractive index of the target. Therefore, indirect manipulation through beads formations [36] or more complex micro-tools [37] have been proposed.

This section demonstrates the capability to use the proposed platform for the indirect manipulation of cells through optical robots. This type of manipulation is a good illustration of the capabilities and versatility of the platform as indirect manipulation through beads is more complex and time-consuming than direct manipulation [38]. Furthermore, controlling the 3D motion of optical robots, where optical handles are rigidly linked, is more demanding than controlling separated and individual beads. Relative deviations in the position of each trap and synchronization problems in the movements of groups will affect the stability of the whole structure.

#### 3.2.1. Fabrication and Collection of the Robots

**Figure 8:**
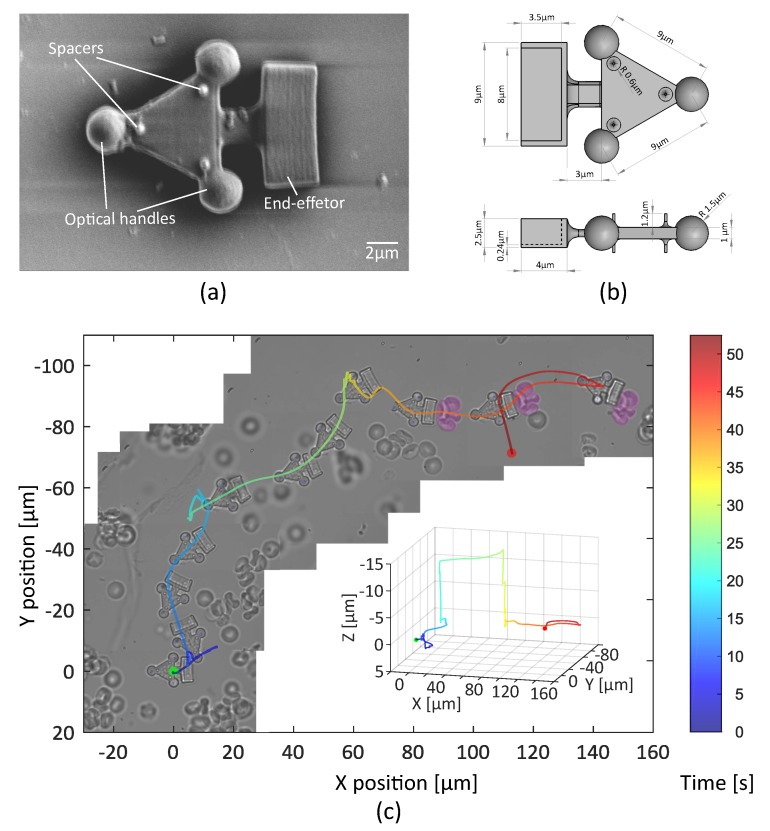
Robot with a shovel-shaped end-effector for cell transport. (**a**) Scanning electron microscopy (SEM) image of the robot. (**b**) Schematic depictions with dimensions of the robot. (**c**) 2D trajectory of the teleoperated robot with an inset of the 3D trajectory. Note that frames between T: 20 s and T: 30 s show the erythrocytes out-of-focus as the robot is elevated in the Z-direction. Then, a cluster of cells (colored in violet) is transported for 80 μm. Results also shown in Supplemental Video S3.

**Figure 9:**
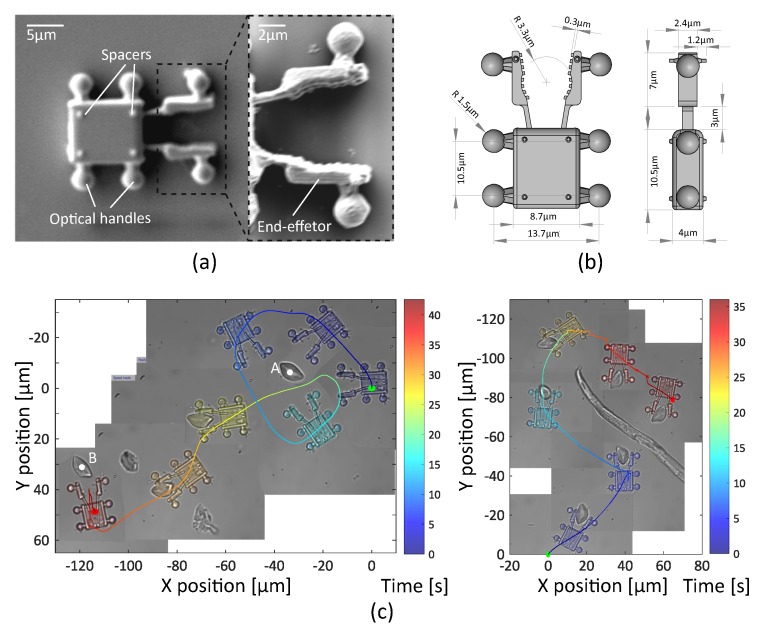
Robot equipped with a fork end-effector for single-cell manipulation. (**a**) Scanning electron microscopy (SEM) image of the robot. (**b**) Schematic depictions with dimensions of optical robot. (**c**) Two sets of experiments manipulation of a single red blood cell and its 2D trajectory. Please see Supplementary Video S4 for complete footage of these experiments.

Two optical robots with three and four spherical handles have been designed. These micro structures are manufactured following dimensional specifications shown in Figure 8b and Figure 9b, by two-photon polymerization (Nanoscribe) using IP-Dip resin (refractive index ∼ 1.52). The first robot has a shovel-shaped end-effector, in order to tow and move several cells at the same time. The second robot has a fork end-effector in order to dexterously manipulate a single-cell. Different spacers are attached on both sides of the robot to minimize the adhesion forces. (See Figure 8a and Figure 9a).

After fabrication, the robots are incubated in a 94.5% distilled water, 5% ethanol and 0.5% *Tween20* solution to prevent the surface adhesion. For experiments, micro-robots are transferred to a sample chamber containing suspended erythrocytes through an actuated microliter syringe (Hamilton, 250 μL). Then, the sample chamber is sealed with a cover-slip, forming a confined environment.

#### 3.2.2. Teleoperated Optical Robot for Cells Transportation

The second experiment consists in the indirect transportation of a cluster of cells. A group of three traps in triangle formation is generated using the user interface. Then, teleoperation is performed through the master device, allowing six-DoF motions.

Thanks to the low response-time and transparency of the system, the operator manages to open a path through a sample, heavily loaded with suspended RBCs. Figure 8c shows the trajectory of the teleoperated Robot.

Initially the robot moves in the microscope-slide bottom (T: 0–19 s). Then, the robot is lifted in the Z-direction (10 μm) as the obstacles completely block the path (T: 20 s). Once the target cells are identified, the axial position of the robot is lowered until it hits the bottom of the slide again (T: 30 s). Then, the robot moves the target cells indirectly (T: 35–45 s). The user dexterously moves the robot through the sample for more than 100 μm to finally displace the cluster of cells for 80 μm. These results can be observed in Supplementary Video S3.

#### 3.2.3. Teleoperated Optical Robot for Dexterous Single-Cell Manipulation

This experiment is dedicated to dexterously manipulate a single-cell. A robot with fork end-effector is designed according to a RBC shape (∼6 μm in diameter) in order to allow full mobility of the manipulated cell. Clamp edges touching the cell are jagged, to limit the contact surface. A group of four traps is used to induce 3D motions.

In the experiments, the cell is seized and manipulated without major difficulties. Experimental results are shown in Figure 9c. In the first task, the optical robot turns around the target, a single RBC located in point A, during an exploration phase (T: 0–20 s). Then, seize the cell and transports it to point B, about 100 μm away between two obstacles (T: 20–39 s). Finally, it deposits the cell to point B moving backwards (T: 39–42 s). The second task consists in manipulating a single cell avoiding a big obstacle. Please see the Supplementary Video S4 for the whole experiment.

During all manipulations, the rotations of the robot have been controlled in position mode. On the other hand, translations have been executed with a mixed control in position and velocity according to the situation. In displacements and cell transportation, the speed control has been preferred, while in contact, loading, and delivery of cells, the control in position has been chosen. The maximal velocity for a four handles optical robot is measured as 60 μm/s for rotation and 100 μm/s for translations. Design differences in optical robots (e.g., number of optical handles, size and shape of the end-effector) will impact the performance of the manipulation. Further investigations in the optimization of shape and size of optical robots, e.g., using simulations, should be needed to take advantage of the full potential of optical robots during indirect manipulation tasks.

Indirect 2D rotation of the cell can be performed around any center point. To achieve complete indirect 3D rotation of a cell, it is necessary to firmly grasp the cell and move it along Z-direction without losing it. This could be accomplished, e.g., by using two separated robots to handle the cell, which will be explored in the near future.

## 4. Conclusions

We proposed and experimentally demonstrated a platform for dexterous cell handling through optical manipulation. As a result of an efficient architecture, the manipulation of more than 15 optical traps in a workspace of (70 × 50 × 9) $\mu m^{3}$ for rotations and (200 × 200 × 200) $\mu m^{3}$ for translations, both with nanometric resolution, is presented. Complex arrangements of optical traps can be grouped and transformed in a variety of ways to achieve diverse tasks.

The characterization of the system shows a static precision of less than 0.4 μm and 1 μm for the manipulation of four and thirteen simultaneous trapped micro-beads, respectively. Evaluation of the stability and efficiency in trajectory tasks shows the suitability of the system for teleoperated control. Concerning the dynamic motion, a threshold velocity has been defined according to the number of traps, in order to assist the user and ensure the retention of trapped micro-beads. The system provides a straightforward human/machine interaction through a tele–robotic solution allowing dexterous manipulation of synthetic and biological objects in an efficient and intuitive way.

Thanks to its capabilities, and because of the stable and simple set-up design, relevant tasks have been demonstrated in a real biological environment with red blood cells. Besides multiple degrees-of-freedom 3D cells rotation, indirect optical manipulations with 3D printed micro tools were also demonstrated. These experiments illustrate the kind of tasks where the presented platform could be implemented. Possible biological applications that can benefit from the proposed platform include cell sorting, isolation, rotation, stimulation, 3D tomographic imaging, and can contribute to more complex tasks such as single-cell surgeries (e.g., nuclear transplantation, embryo micro-injections and polar-body biopsy [39,40]) or micro-assembling [41].

In contrast to other techniques using magnetic or acoustic fields, the high spatial resolution of optical manipulation allows the straightforward implementation of collaborative tasks by several robots and additional degrees of freedom in individual robots. As future work, we plan to expand the platform in order to allow simultaneous control of several optical robots for collaborative tasks and add 3D force-sensing capabilities to these robots, similar to what was presented in [22]. We believe that teleoperated optical robots with force-feedback will be advantageous for numerous biomedical applications leading micromanipulation to a new level of interaction.

## Figures and Tables

**Table 1:** Summary of the system parameters.

Parameter	Value
Teleoperation loop	200 Hz
Simultaneous optical traps	>15
Translation range	200 × 200 × 200 $\mu m^{3}$
Rotation range	70 × 50 × 9 $\mu m^{3}$
Trans Max velocity (1T,4T) *	1500, 110 μm/s **
Rot Max velocity (1T,4T) *	462, 105 μm/s **
Static error (1T,4T) *	<200 nm, 400 nm **

* Depends on the trapped object; ** For 3 μm polystyrene beads.

## Supplementary Materials

The following are available online at https://www.mdpi.com/2072-666X/10/10/677/s1: Video S1: Teleoperated 3D rotation of red blood cell in position mode; Video S2: Teleoperated 3D rotation of red blood cell in velocity mode; Video S 3: Indirect manipulation through a teleoperated optical-robot for cells transportation; and Video S4: Indirect manipulation through a teleoperated optical-robot for single-cell dexterous manipulation.
